# Supplementary material for: Design, Synthesis and Biological Evaluation of Pentacyclic Triterpene Derivatives: Optimization of Anti-ABL Kinase Activity
Source: Molecules. 2019 Sep 30;24(19):3535. doi: 10.3390/molecules24193535 (PMC6804044; doi:10.3390/molecules24193535)
Supplement: Supplementary file 1 [file molecules-24-03535-s001.pdf]

Supplementary Material: NMR Data
Design, Synthesis and Biological Evaluation of Pentacyclic Triterpene Derivatives: Optimization of Anti-ABL Kinase Activity

Halil I. Ciftci, Mohamed O. Radwan, Safiye E. Ozturk, N. Gokce Ulusoy, Ece Sozer, Doha E. Ellakwa, Zeynep Ocak, Mustafa Can, Taha F.S. Ali, Howaida. I. Abd-Alla, Nurettin Yayli, Masami Otsuka*, and Mikako Fujita*

* Corresponding authors

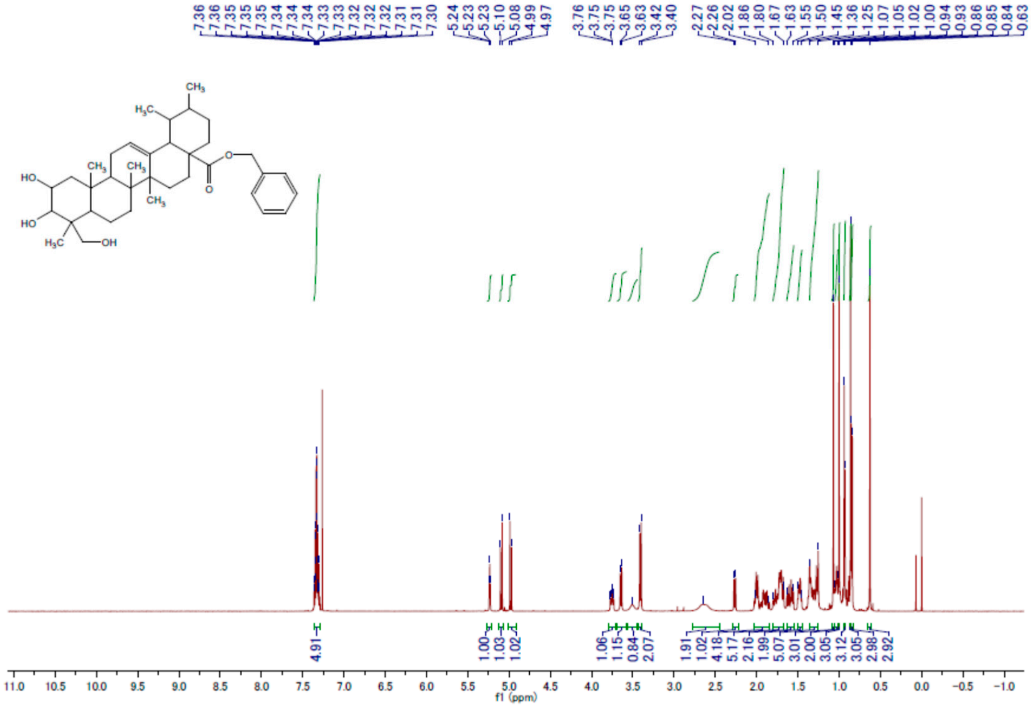

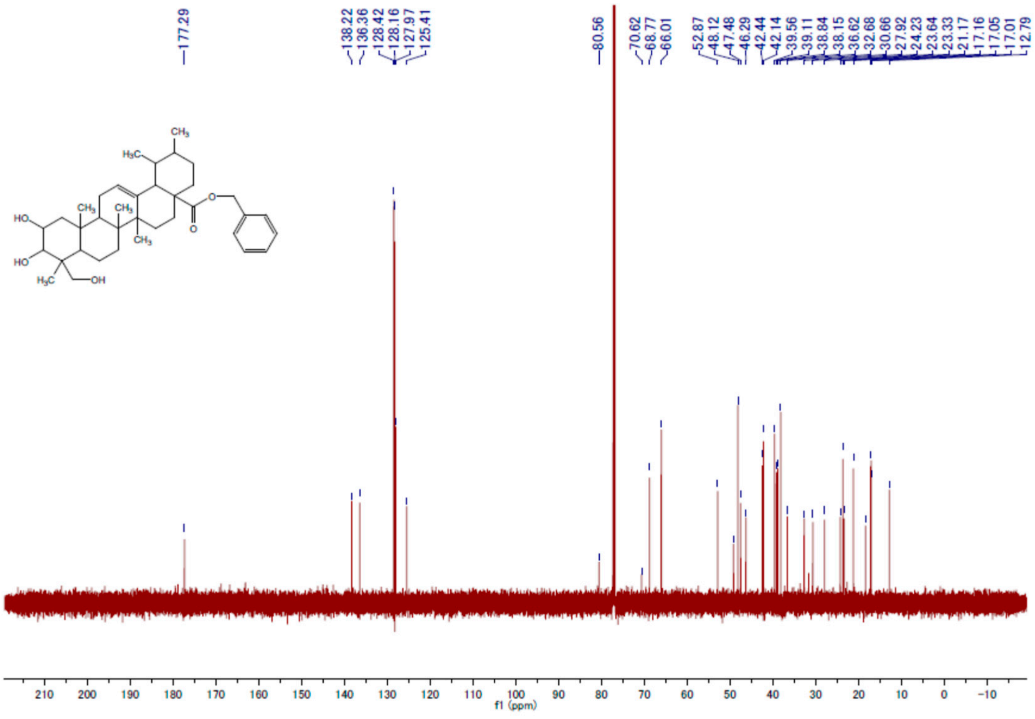

Figure S1: ^{1}H and ^{13}C spectra of **PT1**

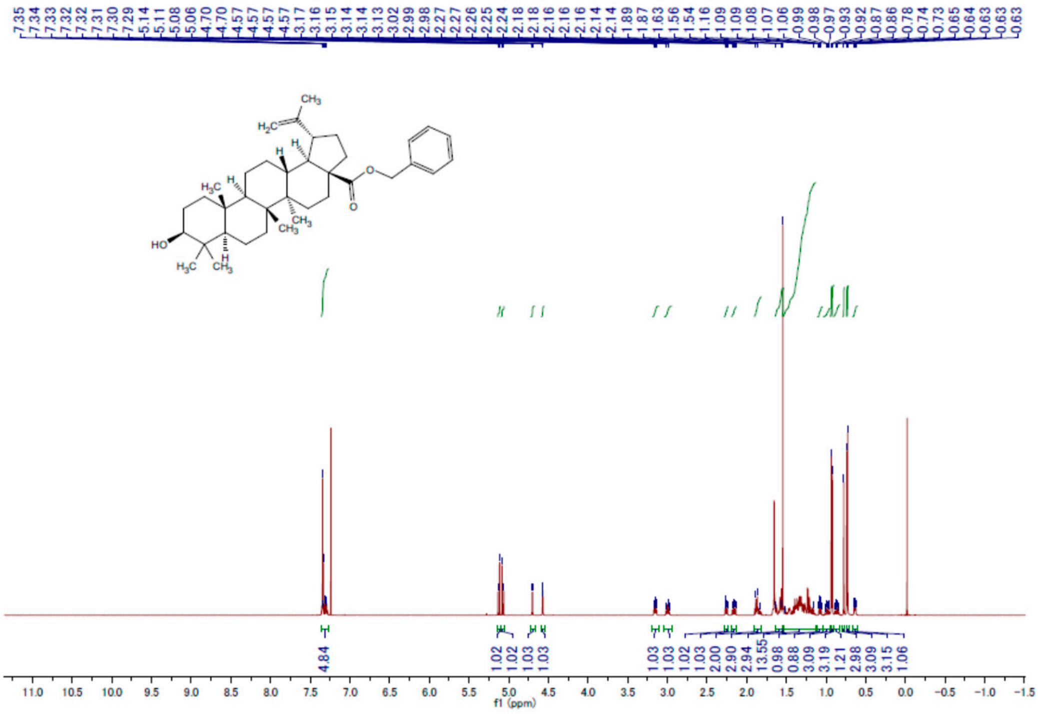

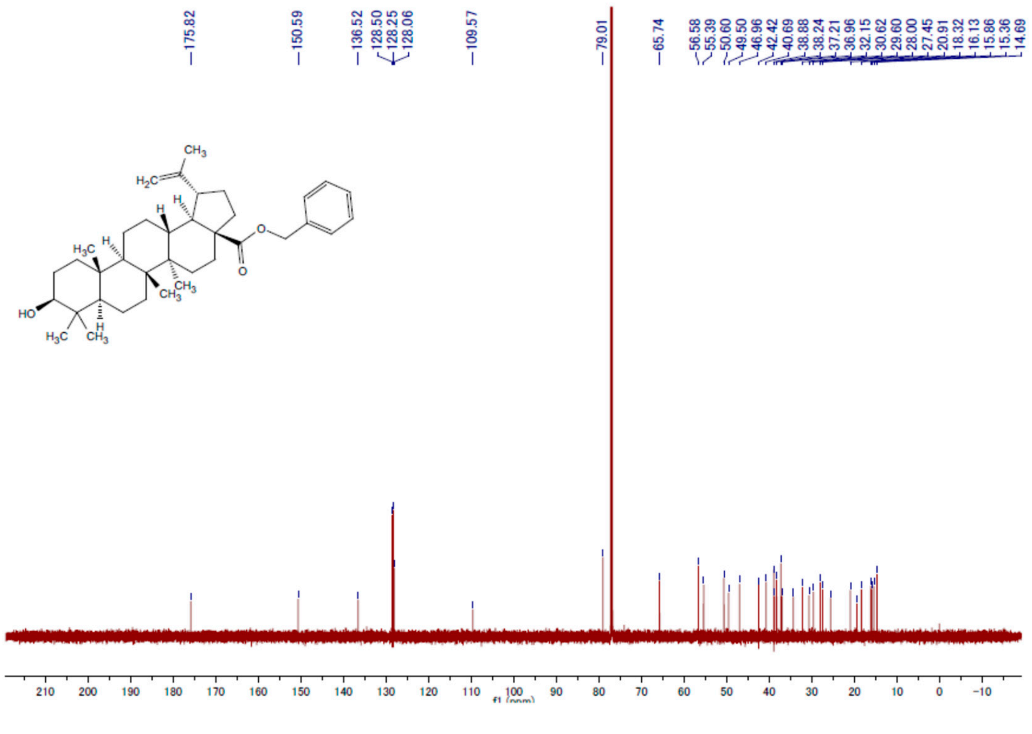

Figure S2: ^{1}H and ^{13}C spectra of **PT2**

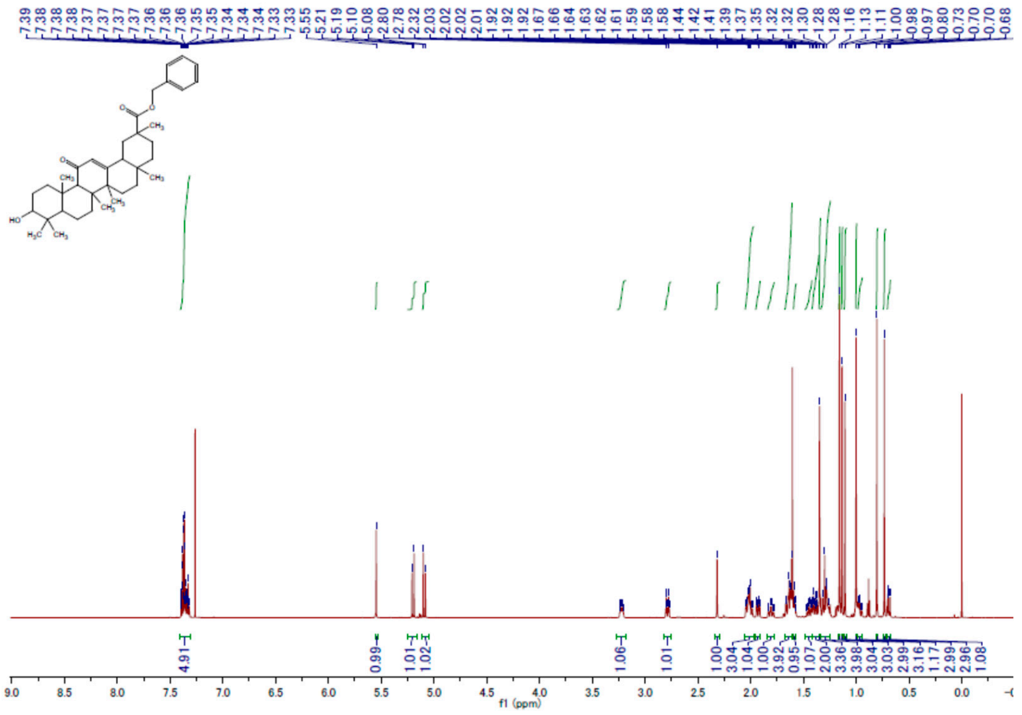

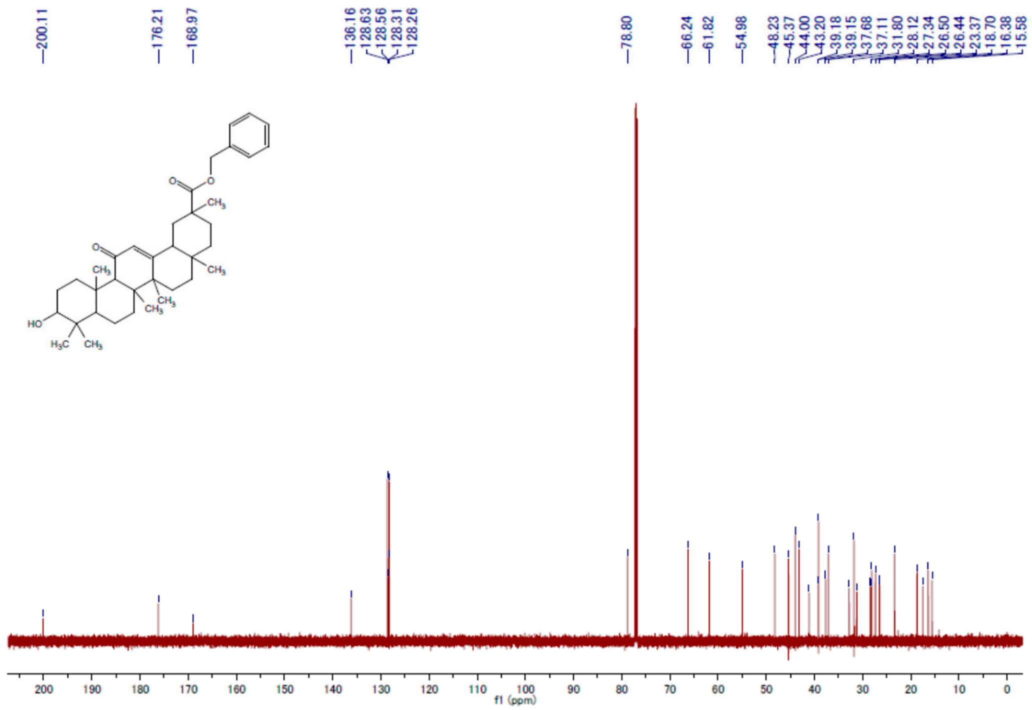

Figure S3: ^{1}H and ^{13}C spectra of **PT3**

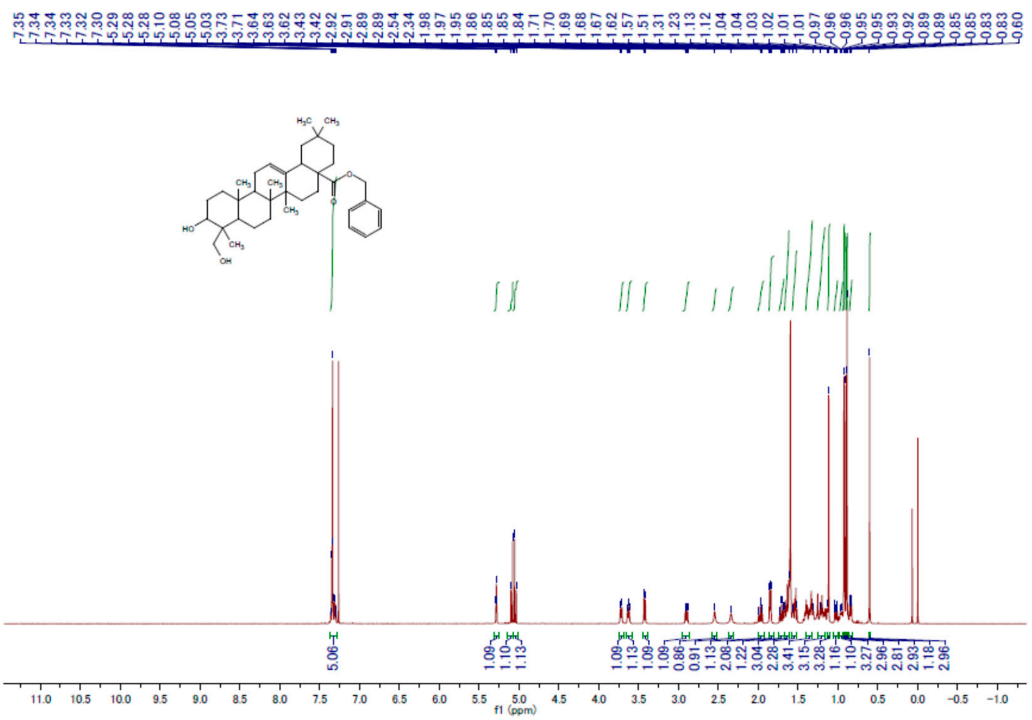

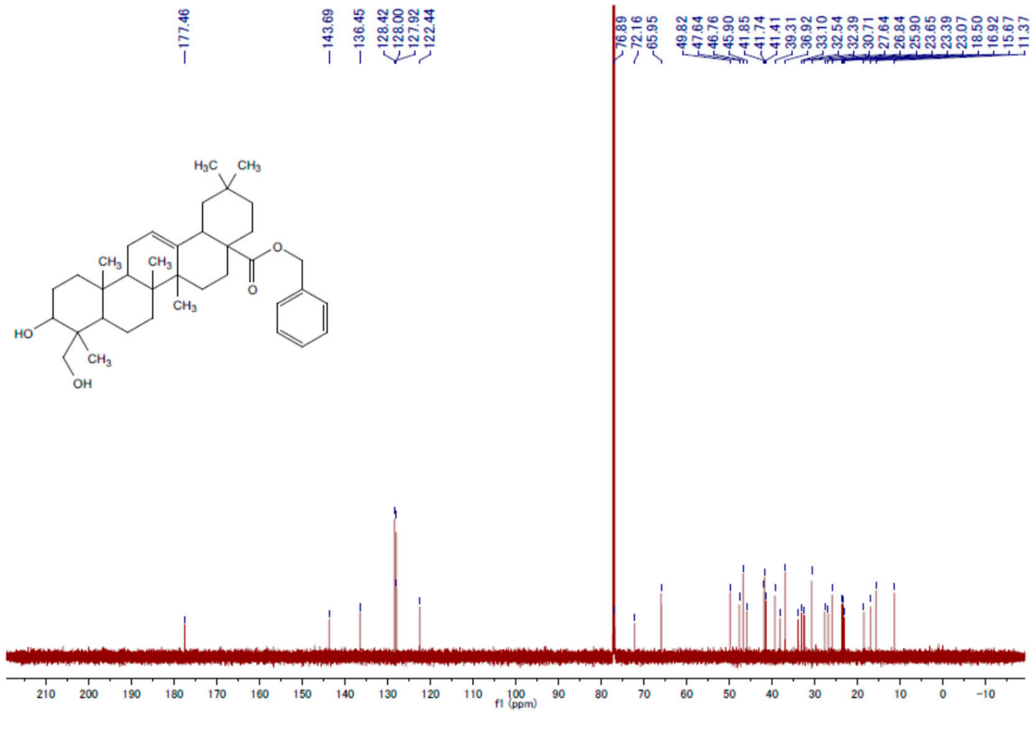

Figure S4: ^{1}H and ^{13}C spectra of **PT4**

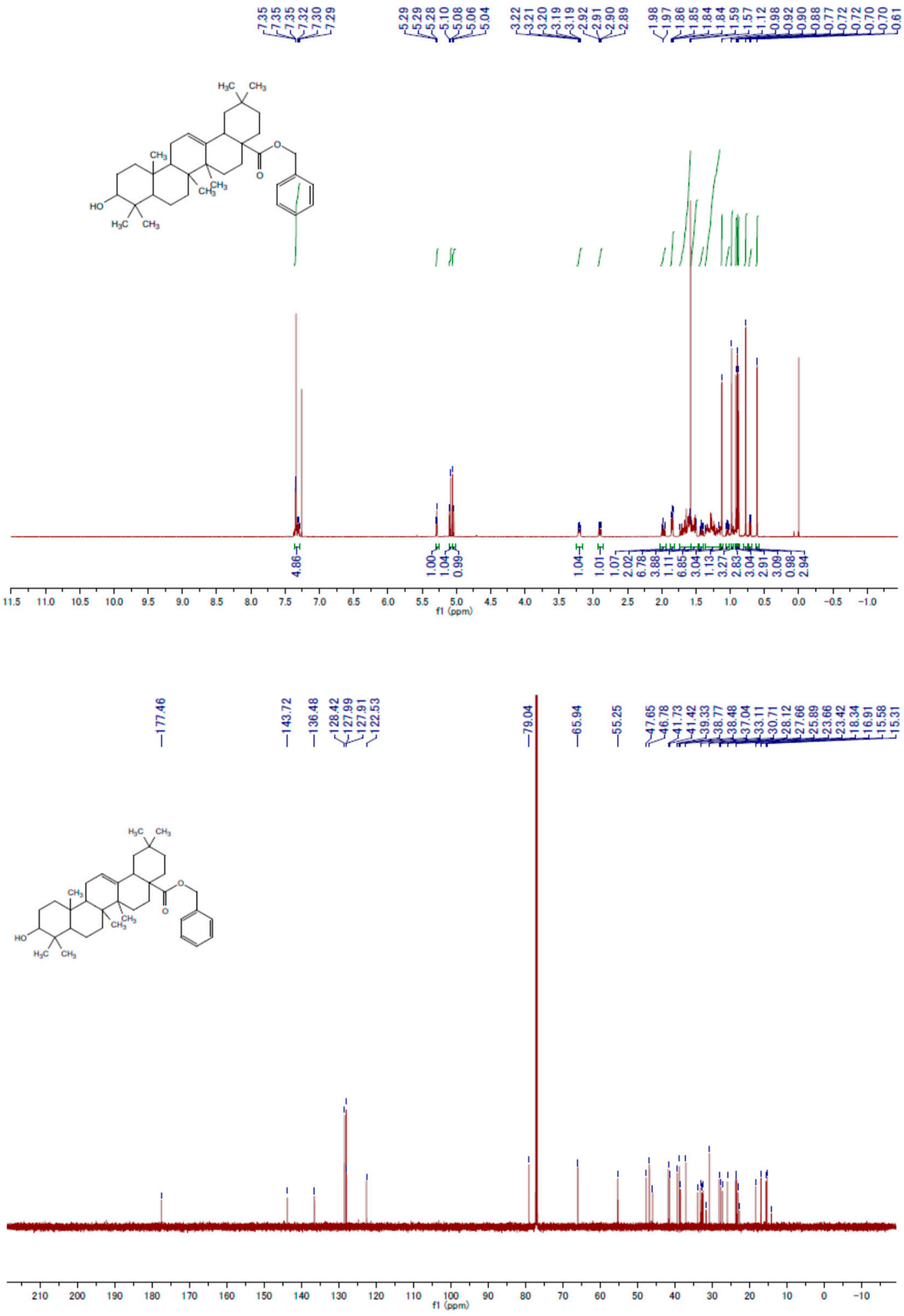

Figure S5: ^{1}H and ^{13}C spectra of **PT5**

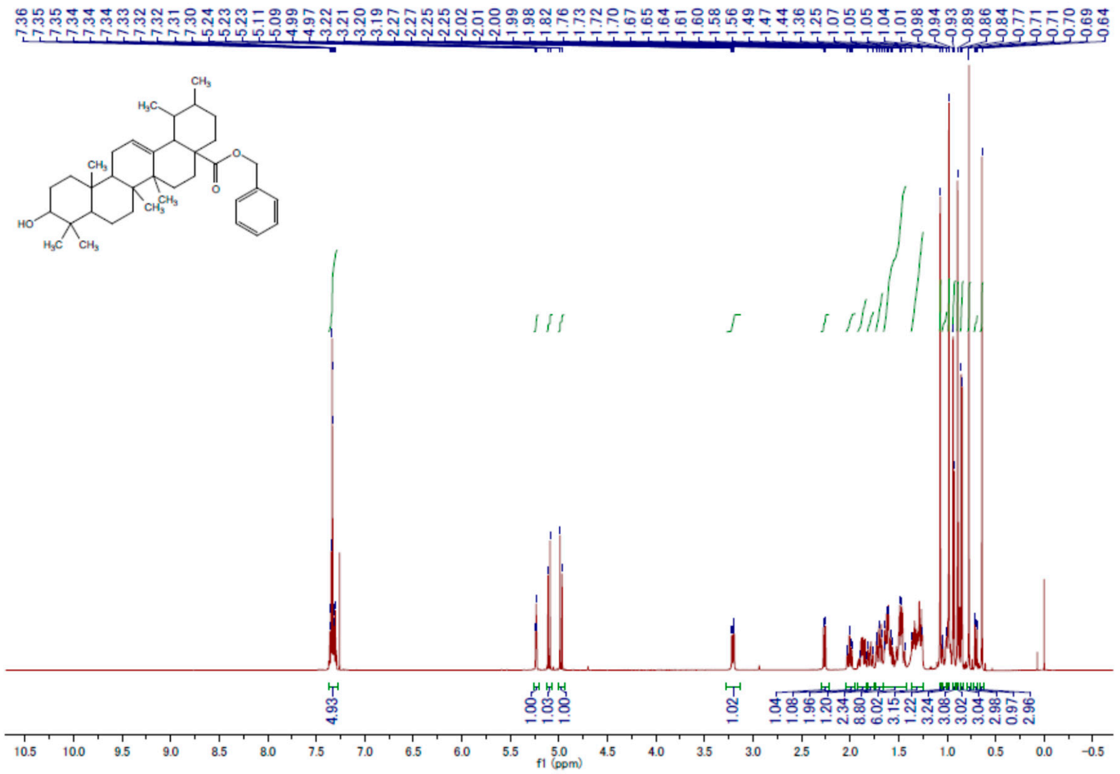

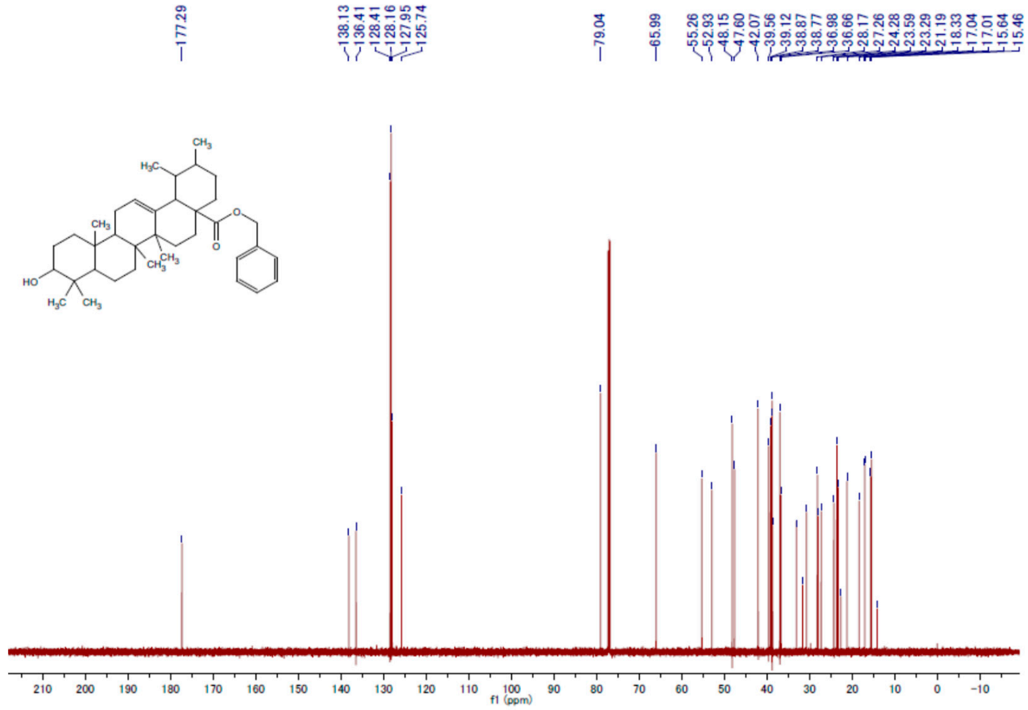

Figure S6: ^{1}H and ^{13}C spectra of **PT6**

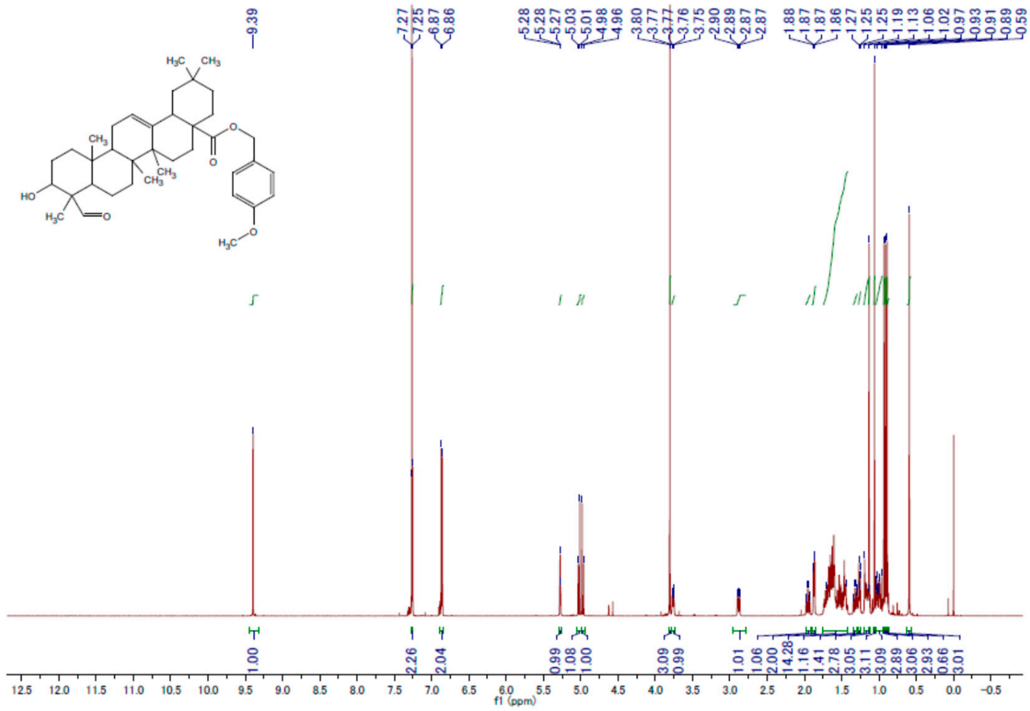

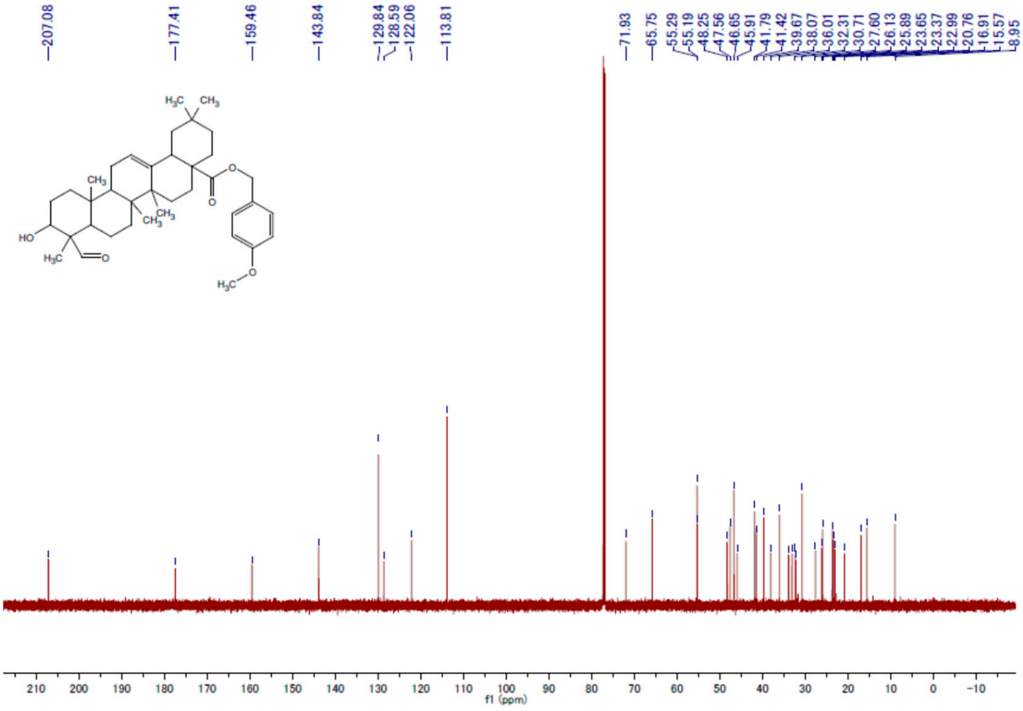

Figure S7: ^{1}H and ^{13}C spectra of **GP1**

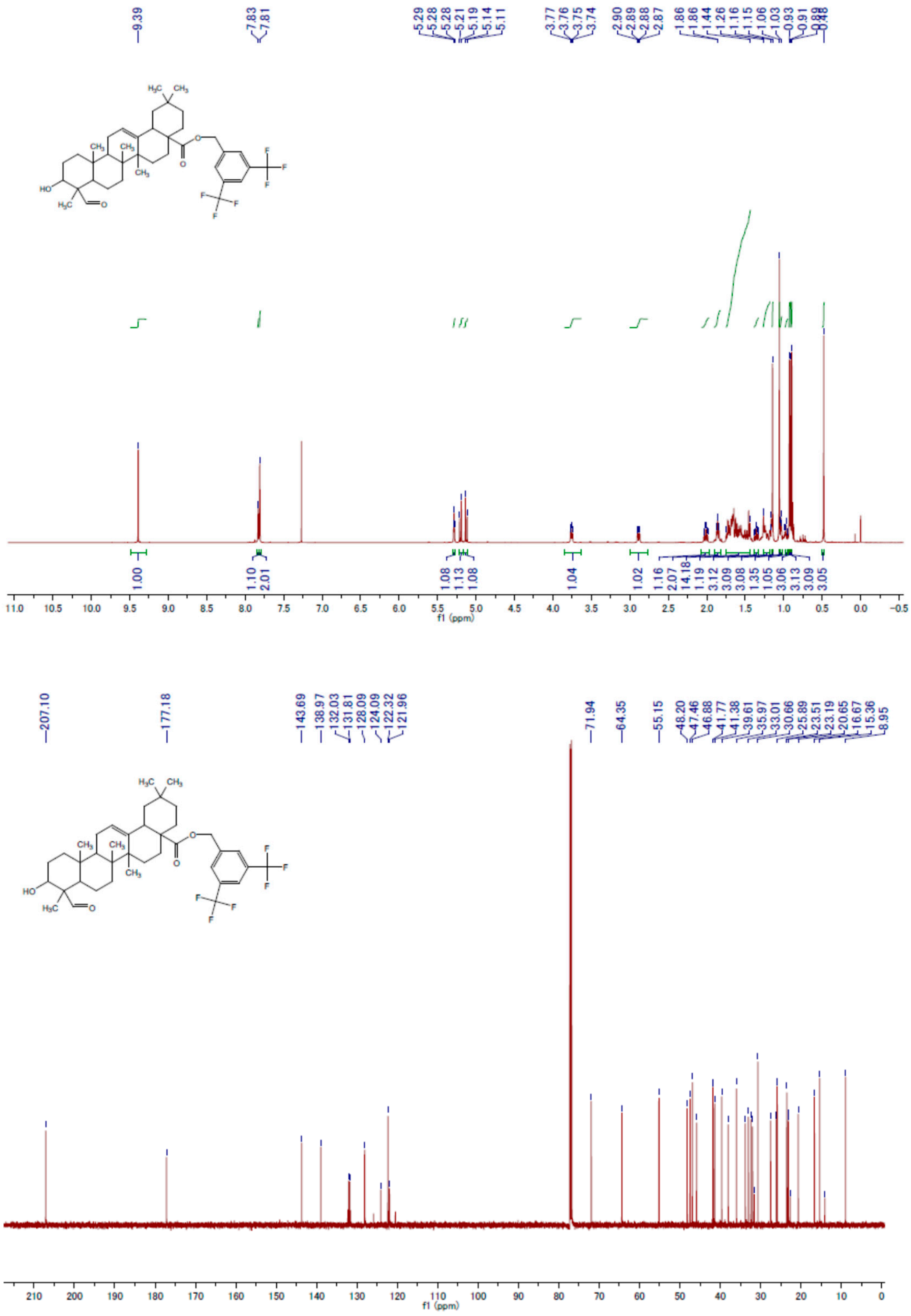

Figure S8: ^{1}H and ^{13}C spectra of **GP2**

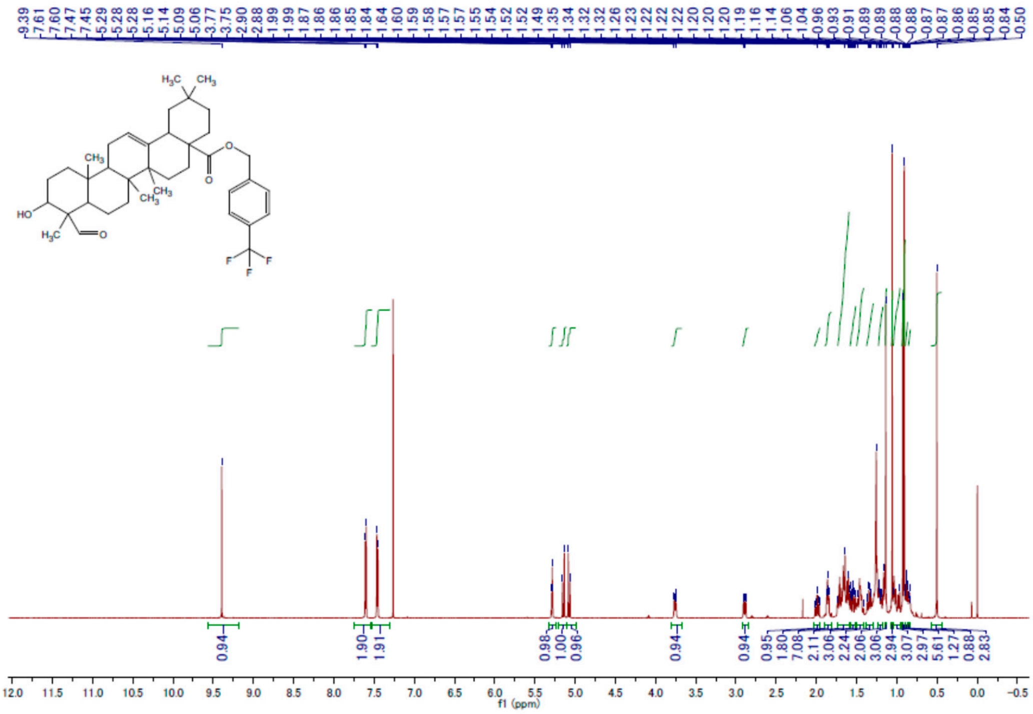

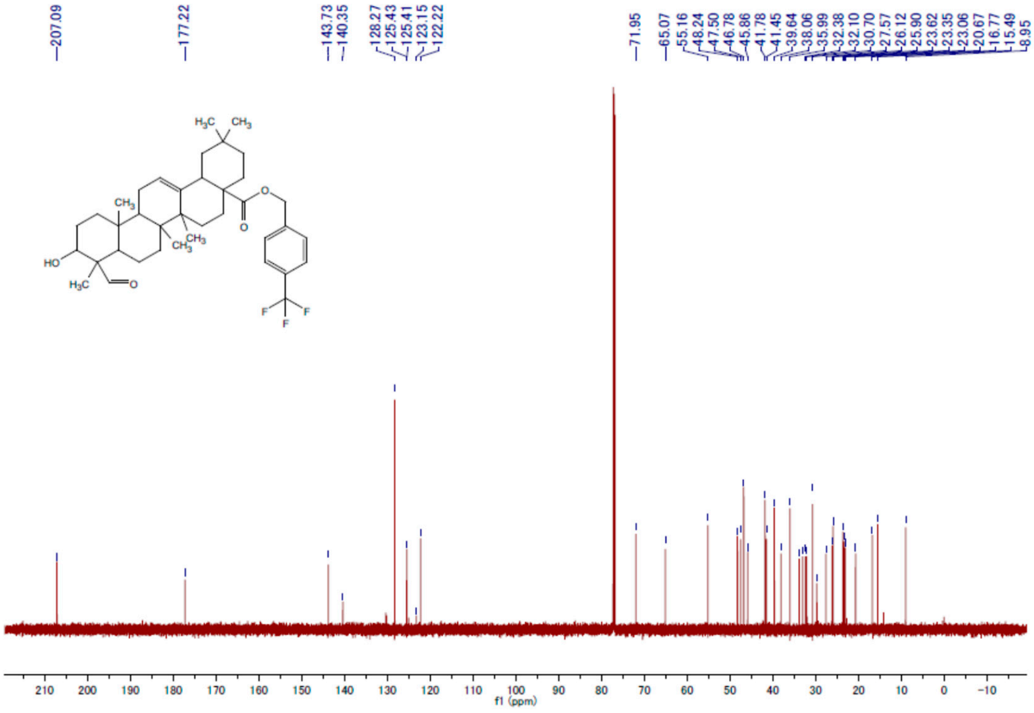

Figure S9: ^{1}H and ^{13}C spectra of **GP3**

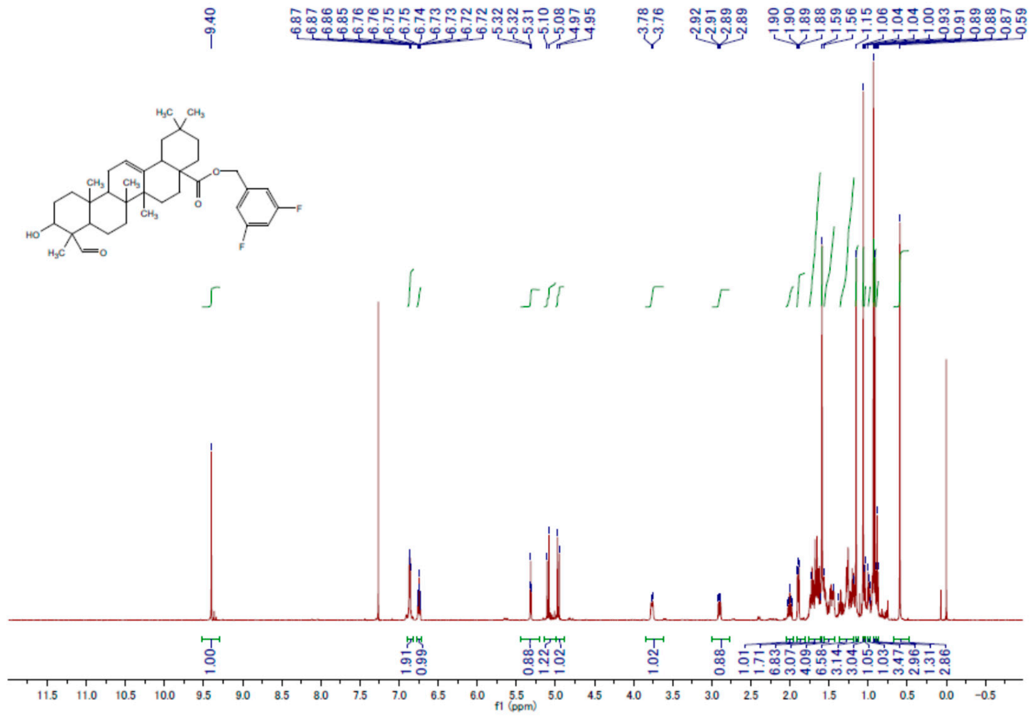

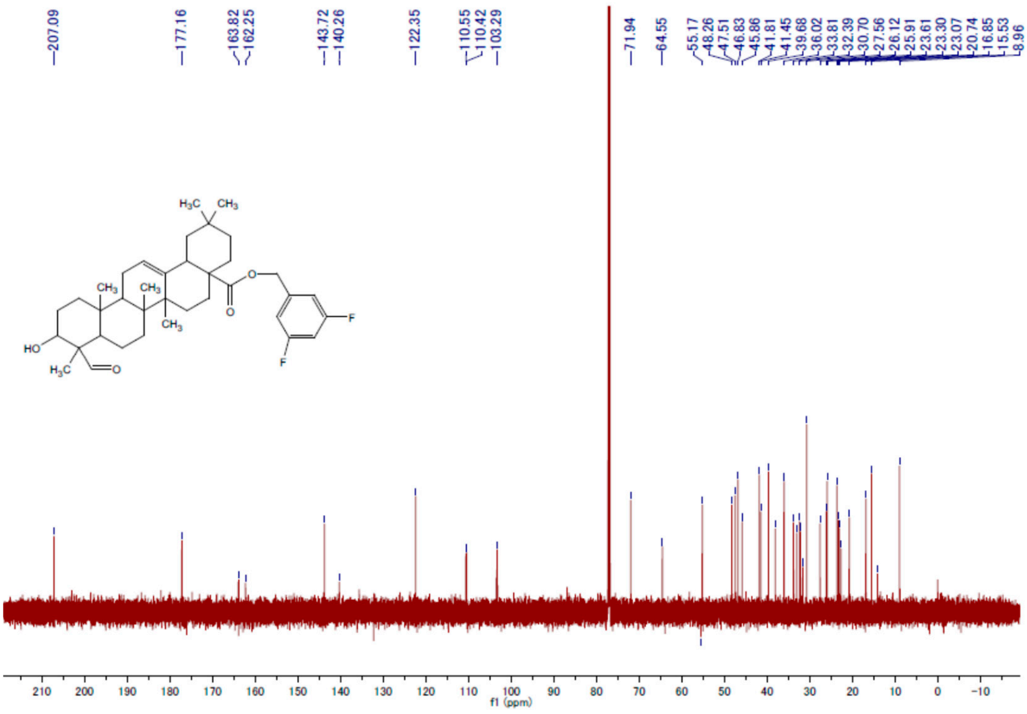

Figure S10: ^{1}H and ^{13}C spectra of **GP4**

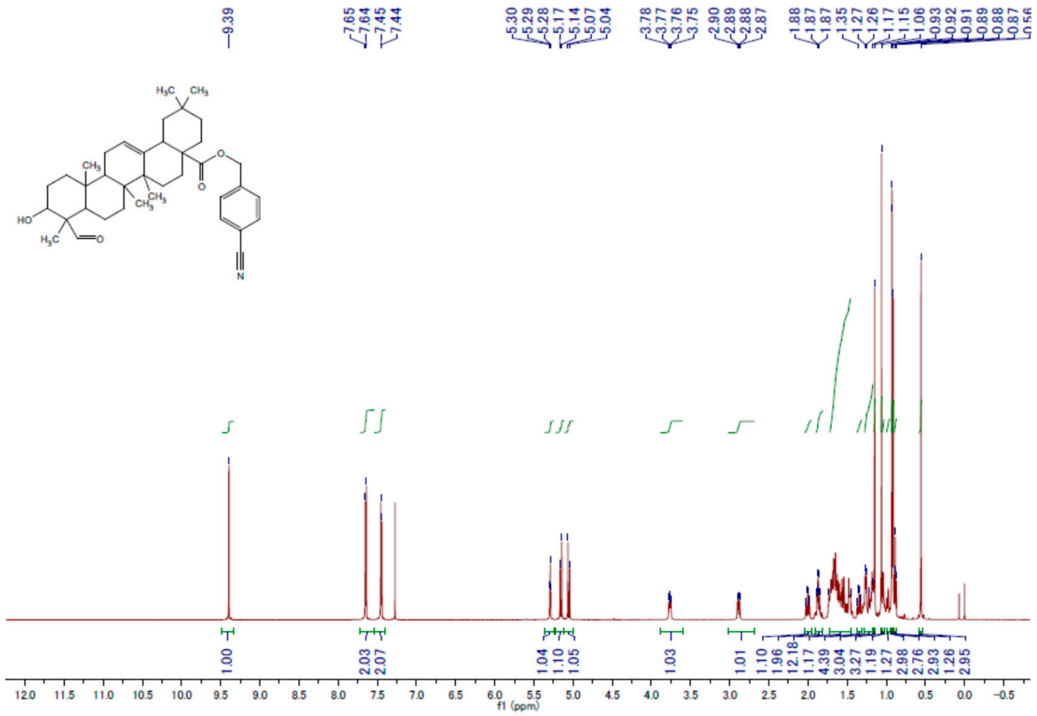

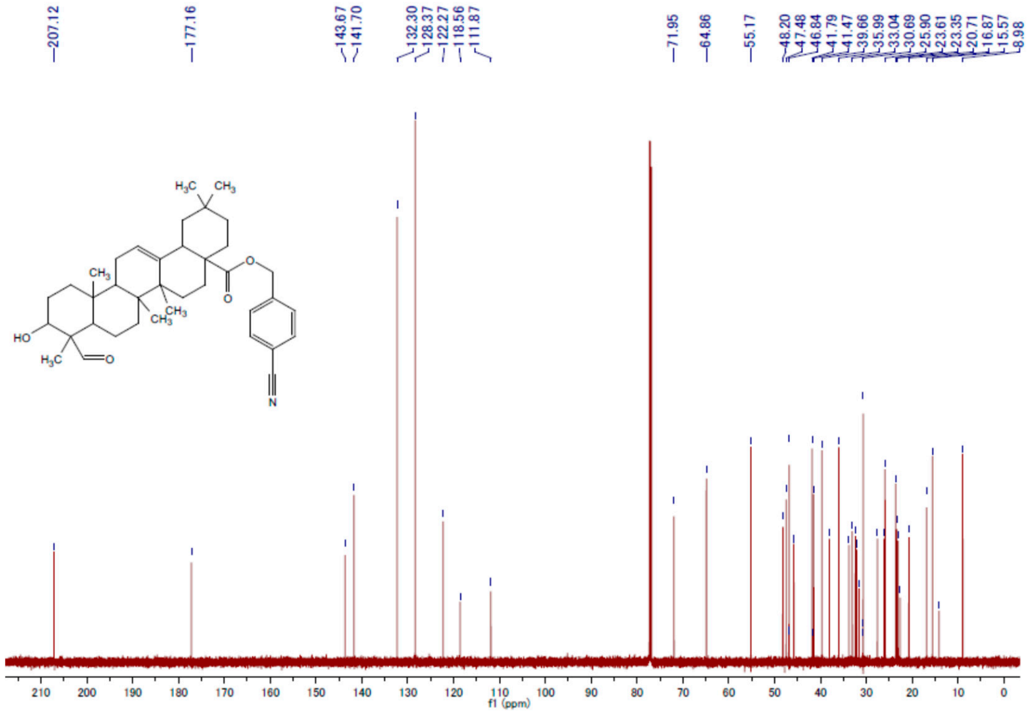

Figure S11: ^{1}H and ^{13}C spectra of **GP5**
